# Rapid and Efficient Screening of *Helicobacter pylori* in Gastric Samples Stained with Warthin–Starry Using Deep Learning

**DOI:** 10.3390/diagnostics15091085

**Published:** 2025-04-24

**Authors:** José Aneiros-Fernández, Pedro Montero Pavón, Natalia García Gómez, Rosa María Palo Prian, Ismael Sánchez García, Ana Isabel Romero Ortiz, Rodrigo López Castro, César Casado-Sánchez, Víctor Sánchez Turrión, Antonio Luna, Manuel Álvaro Berbís

**Affiliations:** 1 Department of Pathology, University Hospital Complex of Granada, 18014 Granada, Spain; janeirosf@hotmail.com (J.A.F.); 2 Department of Anatomical Pathology, Hospital San Juan de la Cruz, 23400 Úbeda, Jaén, Spain; p.montero@cells-ia.com (P.M.P); 3 Cells IA Technologies, 28006 Madrid, Spain; i.sanchez@htmedica.com (I.S.G.);; 4 Department of Histology, Faculty of Medicine, University of Cádiz, 11003 Cádiz, Spain; natalia.garcia@uca.es (N.G.G.); 5 Pathology Department, University Hospital Virgen de las Nieves, 18014 Granada, Spain; rosamaloprian@gmail.com (R.M.P.P.); anaromeroortiz1@hotmail.com (A.I.R.O.); 6 Department of R&D, HT Médica, San Juan de Dios Hospital,14960 Córdoba, Spain; 7 Pathology Department, San Cecilio University Clinic Hospital, 18016 Granada, Spain; rodrigoh.lopez.sspa@juntadeandalucia.es (R.L.C.); 8 Faculty of Medicine, Autonomous University of Madrid, 28029 Madrid, Spain; doctorcasado@hotmail.com (C.C.S.); victor.turrion@uam.es (V.S.T.); 9 Department of Plastic and Reconstructive Surgery, La Paz University Hospital, 28046 Madrid, Spain; 10 Department of General Surgery and Digestive Tract, Puerta de Hierro-Majadahonda University Hospital, 28222 Madrid, Spain; 11 Department of Integrated Diagnostics, HT Médica, Clínica Las Nieves, 23007 Jaén, Spain; aluna70@htmedica.com (A.L.)

**Keywords:** artificial intelligence, gastroenterology, *Helicobacter pylori*, deep learning, whole-slide imaging, digital pathology

## Abstract

**Background/Objectives:*** Helicobacter pylori* is a major risk factor for gastric cancer. The incidence and prevalence of the pathogen are increasing worldwide, urging novel approaches to reduce detection turnaround times. *H. pylori* diagnosis relies on histological examination of gastric biopsies, but interobserver variability considerably impacts its identification. We present an algorithm combining a feature pyramid network and a ResNet architecture for automatic and rapid *H. pylori* detection in digitized Warthin–Starry-stained gastric biopsies. **Methods**: Whole-slide images were segmented into manually annotated smaller patches and segments containing stomach tissue were analyzed for the presence of Gram-negative bacteria. Patches classified as positive were examined to confirm the presence/absence of bacteria in contact with the gastric epithelial surface (*H. pylori*). **Results**: The algorithm exhibited 0.923 average precision and 0.982 average recall. The conducted efficiency study demonstrated that algorithm utilization significantly decreased (*p* < 0.001) diagnostic turnaround times for all participants (two pathologists, a pathology resident, a pathology technician, and a biotechnologist), observing an 88.13–91.76% time reduction. Implementation of the algorithm also improved diagnostic accuracy for the resident, technician, and biotechnologist, indicating that the tool remarkably supports less experienced personnel. **Conclusions**: We believe that the incorporation of our algorithm into pathology workflows will help standardize diagnostic protocols and drastically reduce *H. pylori* diagnostic turnaround times.

## 1. Introduction

*Helicobacter pylori* is a Gram-negative bacterial pathogen characterized by its spiral shape, flagella, large genome, complex antibiotic resistome, robust innate immune system, and intricate innate and adaptive metabolism. In 1983, Warren and Marshall published a paper describing the first-time identification of this bacterium from gastritis biopsy specimens [1] and its pathogenic role in the gastric and duodenal mucosa, receiving the Nobel Prize for their pioneering work in 2005 [2,3]. Generally located in the supra-epithelial mucus layer, in close contact with the lining epithelium of the gastric pits and initial regions of the duodenum [4,5], *H. pylori* infection is the most common causative agent of gastric and peptic ulcers, and a significant cause of duodenal ulcers, gastric malignancies, and biliary cirrhosis, as well as multiple extragastric diseases [6]. Additionally, alongside the colon parasite *Helicobacter hepaticus*, *H. pylori* is responsible for approximately half of all liver cancers [7]. The human stomach is a known reservoir for *H. pylori,* where the pathogen can overcome the highly acidic environment by locally raising the pH via urease-dependent conversion of urea into ammonia and carbon dioxide or by using its flagella to breach the mucosa, thus escaping the gastric lumen [8,9]. Although the presence of *H. pylori* can trigger innate and adaptive immune responses, these are often not enough to overcome the infection, with the pathogen persisting for decades in the absence of treatment [8].

Although *H. pylori* is primarily transmitted through person-to-person contact, environmental contamination, age, and socioeconomic components have also been identified as contributing factors in acquiring the infection [10]. Nowadays, *H. pylori* infection can be found in approximately 50% of the worldwide population, with its incidence in the last few decades having increased due to population growth and ineffective eradication measures [11]. Despite the number of asymptomatic infected individuals being as high as 90%, *H. pylori* infection represents a serious health risk whose eradication has become increasingly difficult due to the gastric pH, bacterial load, compromised mucosa, and the alarming surge in antibiotic resistance observed in recent decades [12,13]. This high prevalence has led to the development of multiple diagnosis methods for the detection of the pathogen, including non-invasive (e.g., the urea breath test, presence of specific antigens in the stools, serology) and invasive techniques that require an endoscopy and/or biopsy (histological examination, culture, rapid urea test, polymerase chain reaction (PCR) test) [14]. Among these, histopathological examination remains the gold standard for *H. pylori* diagnosis, as it not only allows the detection of the pathogen but also the identification of the many histological alterations caused by the infection [15].

The presence of *H. pylori* in gastric biopsy sections can be determined through staining techniques such as hematoxylin and eosin (H&E), Giemsa, and Warthin–Starry (WS); however, none of them are entirely specific to *H. pylori*, and interpretation can be challenging [16]. While H&E and Giemsa are commonly used, WS is often considered more specific (with a specificity of 90–100%) [15] and provides better contrast for certain bacterial morphological features. However, WS staining can be laborious to perform, highlighting the potential benefits of an automated approach. The utilization of immunohistochemistry (IHC) approaches offers increased sensitivity and specificity over more traditional stains [17], making it a more precise detection method. However, as its routine use is limited by its high cost [18], it is currently employed only in active chronic gastritis cases in which *H. pylori* has not been detected in routine histochemical stains, when the bacterial load is very low, or when it is present in atypical locations [19,20]. Factors such as interobserver variation and the presence of organisms other than *H. pylori* might affect the diagnosis [21], and histopathological examination can offer false negative results due to the irregular distribution of the infection, the lack of a specific stain, an inexperienced pathologist, or the low resolution/quality of the images, as well as false positives due to misdiagnosis of other bacteria [22].

In the last decade, artificial intelligence (AI) has boomed as a field focused on the automatization of intellectual tasks usually performed by humans. In hospital and clinical settings, AI is expected to help standardize and make clinical workflows swifter and more cost-effective, as well as to contribute to more efficient, personalized medical care and more accurate diagnoses [23,24,25]. Specific methods to achieve this level of automation, such as machine learning and deep learning (DL), are applied to tasks requiring high-level pattern recognition, e.g., image classification [26]. The application of AI in healthcare is expanding rapidly, offering powerful tools for the analysis of complex medical data and improving patient outcomes. A recent study evaluating antibiotic resistance employed AI-driven models to analyze resistance patterns in *Mycobacterium tuberculosis*, providing insights that can guide more effective treatment strategies [27]. Additionally, AI has been applied for time-series prediction in medical settings, where a combination of Bayesian approaches and DL improved the forecasting of disease progression and patient monitoring, leading to better clinical decision-making [28]. These applications foresee the potential of AI to enhance disease management and precision medicine.

Here, we present CS-Bacter, a new DL algorithm for the automatic detection of *H. pylori* in WS-stained gastric specimens that facilitates pathological diagnosis by identifying potential *H. pylori* hot spots. Utilization of this algorithm reduced diagnostic turnaround time by up to 91.76%, increased the accuracy of the diagnosis, and facilitated the determination of uncertain cases. For these reasons, we think that our algorithm will be a valuable addition and a welcomed implementation in the clinical workflows of pathology laboratories in the short term.

## 2. Materials and Methods

### 2.1. Algorithm Design and Development

**Figure 1:**
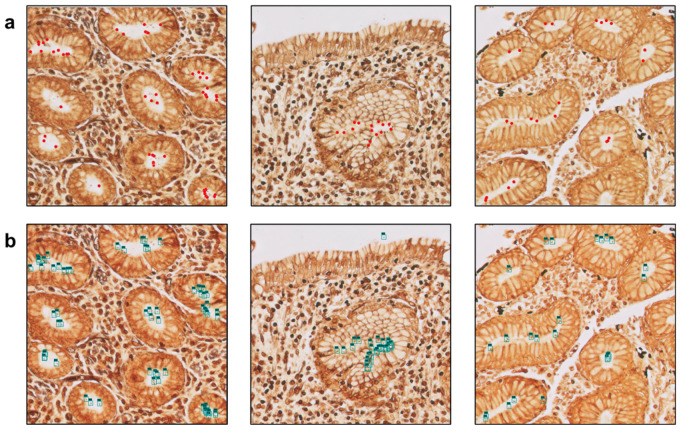
Comparison between manual and algorithm-based bacterial identification (40× magnification). (**a**) The images on the upper row were manually annotated by expert pathologists, with identified bacteria marked as red dots. (**b**) The lower row shows the same images processed by the algorithm, with detected bacteria highlighted with blue bounding boxes.

The CS-Bacter algorithm aims to facilitate the diagnosis of *H. pylori* in WS-stained images from gastric biopsies via the identification of hot-spot areas within the digital image to increase the efficiency and accuracy of the diagnosis using state-of-the-art DL techniques. The algorithm was developed using Python 3.9.0 (www.python.org) and training and testing were conducted on a high-performance computing system equipped with an AMD EPYC CPU (Advanced Micro Devices, Inc., Santa Clara, CA, USA), 192 GB of RAM, and four NVIDIA TITAN RTX GPUs (NVIDIA, Santa Clara, CA, USA). Development of the tool proceeded as follows: (1) analysis of potential solutions for the automatic identification of *H. pylori* hot-spot areas via AI approaches; (2) image annotation for the training of the algorithms—for this, each whole-slide image (WSI) was divided into patches of 1024 × 1024 pixels, which in turn were manually annotated by expert pathologists who identified and localized individual *H. pylori* bacteria within the image patches, marking them with red dots over each one (Figure 1), and selecting both altered (presence of *H. pylori*) and not altered (absence of *H. pylori*) areas (confidence threshold = 0.90), thus classifying the patch as positive (contains *H. pylori*) or negative (rest of the image that does not contain *H. pylori*); (3) exportation of the annotated coordinates of the hot-spot areas for the *H. pylori* detection algorithm; (4) training of the CS-Bacter algorithm.

**Figure 2:**
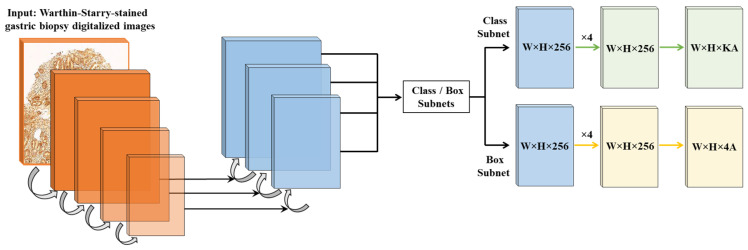
The CS-Bacter algorithm uses a feature pyramid network [32] and a ResNet [31] architecture with two sub-networks attached to this backbone: one for classification (in the image, class subnet) and one for regression (box subnet) to ground truth.

Both the classification neural network EfficientNet [29] and the segmentation network EfficientNet-UNet [30] were considered as potential architectures for our model. After thorough evaluation, we ultimately determined that ResNet [31] provided a better fit for our specific objectives, based on its strong feature extraction capabilities and robust performance in classification tasks. The final model (Figure 2) employs a feature pyramid network [32] and a ResNet [31] architecture adapted to the histopathology domain, with two sub-networks attached to this backbone: one for classification and one for regression. Key hyperparameters are summarized in Table 1. A data augmentation strategy was employed, including rotations, horizontal/vertical flips, and color variations to enhance model robustness.

**Figure 3:**
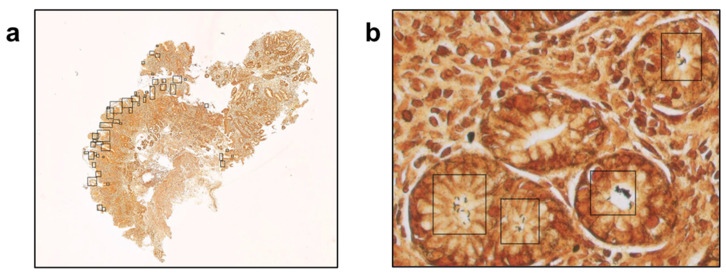
The algorithm efficiently detected the presence of bacteria in Warthin–Starry-stained gastric samples at a 1× magnification (**a**). Observation at a higher magnification (40×) showed that the identified bacteria were all located at the glandular component of the stomach tissue sample (**b**).

The final algorithm (Figure 2) utilizes as input data WSIs of WS-stained gastric samples which are segmented into smaller patches containing stomach tissue; these patches are analyzed for the detection of Gram-negative bacteria, which will be further analyzed to differentiate between bacteria contacting the glandular epithelium (*H. pylori*) [8] and bacteria located outside the glandular component (Figure 3).

**Figure 4:**
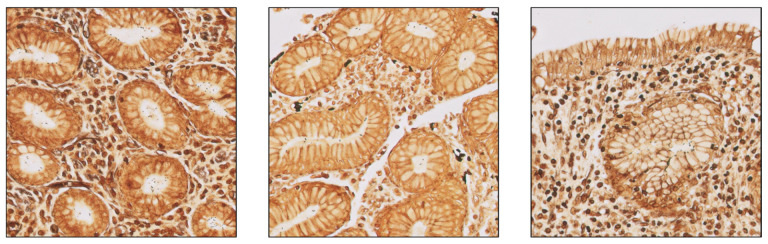
Examples of 1024 × 1024-pixel patches employed to train the algorithm (40× magnification).

The dataset utilized for the development of the algorithm (Table 2) comprised 3300 specimens collected at the HT Medica anatomic pathology centers (Granada, Spain), scanned using a Philips Ultra-Fast Scanner (Philips, Amsterdam, The Netherlands) at 40× magnification, and randomly selected into the different subsets (training, validation, and calibration) by expert pathologists to guarantee maximum variability to make the algorithm more robust. Of the 3300 specimens, 2640 samples were used for algorithm training (Figure 4), 528 for validation, and 132 for calibration (i.e., establishing a threshold that optimizes both accuracy and recall). Different confidence thresholds were analyzed, based on precision and recall, to determine the best value to optimally balance these metrics. For CS-Bacter, prioritizing precision minimized false positives, ensuring reliable detections, though at the risk of missing some bacteria.

### 2.2. Efficiency of AI-Assisted vs. Traditional Digital Pathology Diagnosis

The protocol followed in this study was approved by the Ethical Committee of Biomedical Research of Granada (Comité Ético de Investigación Biomédica de Granada). Written informed consent was obtained from all patients at the time of the performance of the imaging study. This research was performed in accordance with the ethical standards in the 1964 Declaration of Helsinki.

To test the efficiency of the algorithm, a study was performed comparing unaided vs. AI-assisted *H. pylori* diagnostic turnaround time for digitalized WS-stained gastric biopsy samples. The study included two gastrointestinal (GI) pathologists with more than 15 years of experience, a fourth-year pathology resident, a pathology technician with more than ten years of experience, and a biotechnologist with three years of experience performing genetic tests at an anatomic pathology unit. All samples were independently validated by a third GI pathologist with more than 35 years of experience (reference pathologist).

For the calibration of the CS-Bacter algorithm to specifically carry out the designed efficiency study, an independent set consisting of 100 random cases were chosen, and from these, a sub-set of 20 was further selected to carry out an iterative process using groups of five images picked from those 20 for parameter adjustment. The results were subsequently tested on the remaining 80 images (Table 2).

Both the technician and the biotechnologist received specific training to learn to distinguish between *H. pylori*-positive and -negative samples. First, they were provided with relevant literature describing histopathological *H. pylori* detection to ensure consistency with established methodologies. Subsequently, they reviewed a dataset of 100 *H. pylori*-positive and 100 *H. pylori*-negative samples—extracted from the algorithm training dataset (Table 2)—prior to the execution of the efficiency study. During training, our reference pathologist provided them with guidance in cases of uncertainty, ensuring that their identification of *H. pylori* aligned with expert clinical judgment. While no formal clinically accepted grading standard was explicitly used for this training, the process was designed to adhere as closely as possible to real-world histopathological assessment practices.

A total of 300 WSIs of WS-stained gastric samples were selected to conduct the efficiency study, of which 150 (50%) were *H. pylori*-positive and 150 (50%) were *H. pylori*-negative (Table 2). This independent set of images had not been previously employed at any stage of the algorithm development process. The gastric biopsy samples were collected at the Anatomic Pathology Inter-Center Provincial Unit of Granada (Unidad Provincial Intercentros de Anatomía Patológica de la Provincia de Granada, UPIGAP), an inter-Center laboratory that processes and analyzes samples coming from the San Cecilio University Clinic Hospital, the Virgen de las Nieves University Hospital, the Basic General Hospital Baza, and the Basic General Hospital Santa Ana de Motril, all of them located in Granada (Spain), between the months of July and October, 2021. WSIs of WS-stained samples were obtained with a Philips Ultra-Fast Scanner at 40× magnification. Each of the 5 participants analyzed 60 anonymized samples, up to a total of 300 samples. All participants used the same color-calibrated medical-grade 27-inch monitor (2560 × 1440 resolution) and performed the analysis under controlled lighting to minimize variability from display and ambient conditions.

**Figure 5:**
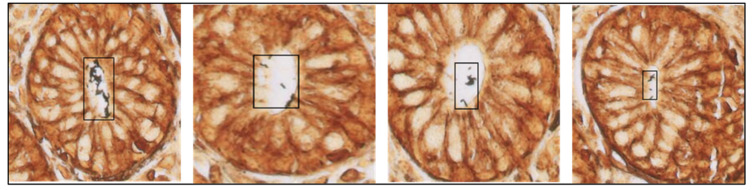
*H. pylori*-positive patches were detected by the algorithm on four different Warthin–Starry-stained gastric samples (40× magnification).

Finally, unaided vs. AI-assisted diagnostic turnaround times were measured and recorded for all the participants (Table 3). To assess whether AI assistance was particularly effective in *H. pylori*-positive or -negative samples, we also recorded turnaround times stratified by *H. pylori* infection status. For the unaided diagnosis, each participant independently analyzed 60 *H. pylori*-positive and -negative randomized WSIs; after a waiting time of 60 days, the same 60 images were newly randomized, uploaded to a sample viewer tool, and presented again to the participants for AI-aided diagnosis. This assistance consisted of the detection and identification of *H. pylori* hot spots by CS-Bacter, which were highlighted by a black box over the image for user verification (Figure 5). The gold standard for this study was established based on the assessment of our reference pathologist, who independently reviewed all cases and determined the presence or absence of *H. pylori* based on histopathological examination of the samples, serving as the ground truth for our analysis. None of the participants enrolled in the study were involved in the generation of this ground truth, ensuring an unbiased evaluation of unassisted vs. AI-assisted diagnosis.

### 2.3. Statistical Analysis

The precision and recall of the CS-Bacter algorithm were calculated using Excel (Microsoft Corporation, Redmond, WA, USA), with an intersection over union (IoU) threshold of 0.5. Precision was calculated as the difference between the number of true positive diagnoses and the sum of true and false positives. Recall was calculated as the difference between the number of true positive diagnoses and the sum of the number of true positives and false negatives.

We also performed a statistical comparison of diagnostic turnaround times with and without AI assistance. All data were checked for normality and analyzed using a paired *t*-test (if normally distributed) or a Wilcoxon signed-rank test (if non-normally distributed). In all cases, *p* < 0.001 was considered significant.

Additionally, we compared the diagnostic accuracy (i.e., the proportion of correct classifications relative to the independent final judgment provided by an expert pathologist) for each participant, with and without AI assistance. Cases of diagnostic discrepancy between participants and the reference pathologist were recorded.

## 3. Results

### 3.1. The Algorithm Can Automatically Detect H. Pylori on Gastric Samples

Histopathological examination remains the gold standard for the diagnosis of *H. pylori*. The CS-Bacter algorithm was designed and developed to facilitate the work of the pathologist by identifying *H. pylori*-positive areas on digitalized WS-stained gastric samples, automatically detecting potential *H. pylori* hot spots so that a swifter, more efficient diagnosis can be made. Identification of these hot-spot areas occurred in two steps: first, the algorithm divided the image into smaller patches and detected the presence of Gram-negative bacteria in the WSI, followed by identification of those bacteria in contact with the gastric epithelial surface (the location of *H. pylori*) (Figure 5). The algorithm was trained on a set of 2640 samples (184,000 patches), manually annotated by expert pathologists, and validated on a set of 528 WSIs, showing a strong performance (IoU threshold = 0.5, average precision = 0.923, average recall = 0.982). The considerable size of the training dataset allowed for the algorithm to be barely influenced by lower-quality stains or samples.

### 3.2. Utilization of the Algorithm Drastically Reduces Diagnostic Turnaround Time

Once the CS-Bacter algorithm was calibrated with the 100-sample dataset collected at the UPIGAP, we analyzed its efficiency and the impact of its implementation in the pathology workflow by comparing diagnostic turnaround time with and without the aid of the algorithm (Table 3). We recruited two pathologists, a pathology resident, a pathology technician, and a biotechnologist to test the efficiency of the algorithm by analyzing 60 digitalized gastric samples, including *H. pylori*-positive and -negative samples, each with and without the assistance of the algorithm. Both the pathology technician and the biotechnologist were trained in the identification of *H. pylori* in gastric samples prior to enrollment.

**Figure 6:**
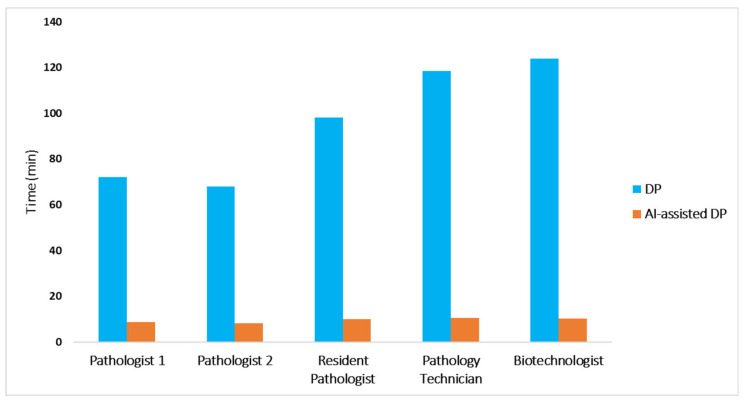
Turnaround digital pathology diagnosis time with (orange) and without (blue) the aid of the algorithm. Use of the CS-Bacter algorithm drastically reduced turnaround times by 88.13–91.76% (*p* < 0.001). DP—digital pathology; AI-assisted DP—artificial intelligence-assisted digital pathology.

Overall, turnaround times were significantly reduced (*p* < 0.001) when using AI assistance. The utilization of the CS-Bacter algorithm contributed to drastically reducing diagnostic turnaround time for all individuals participating in the study (Figure 6): from 71.97 to 8.53 min (88.14% reduction) for pathologist #1; from 67.95 to 8.07 min (88.13% reduction) for pathologist #2; from 98.18 to 9.83 min (89.98% reduction) for the pathology resident; from 118.38 to 10.53 min (91.10% reduction) for the pathology technician; and from 123.95 to 10.22 min (91.76% reduction) for the biotechnologist. Furthermore, when examining turnaround times separately for *H.* pylori-positive vs. -negative samples, we observed that the benefit of AI assistance was slightly greater in *H. pylori*-positive images. This is likely due to the ability of CS-Bacter to directly highlight the bacteria, thus saving time spent searching the WSI.

Additionally, we analyzed whether the use of the algorithm impacted diagnostic accuracy. Compared to our independent expert pathologist, without AI, the overall diagnostic accuracy was 98.3% for pathologists #1 and #2, 95.0% for the pathology resident, 93.3% for the pathology technician, and 94.2% for the biotechnologist. With AI assistance, accuracies rose to 100% for both pathologists and to 98.3% for the pathology resident, the pathology technician, and the biotechnologist (Table 4).

**Figure 7:**
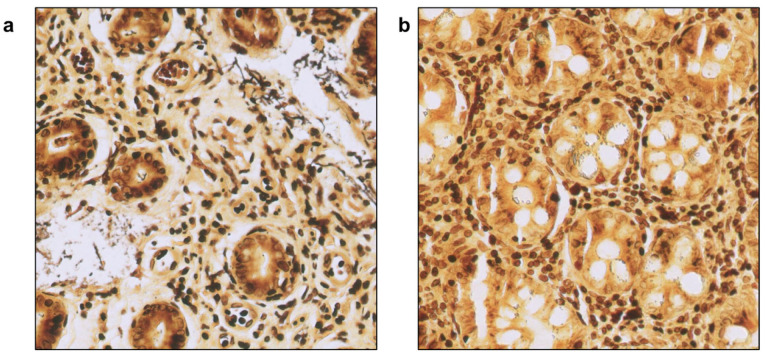
Examples of images that led to discrepancies between unassisted and AI-assisted diagnoses (40× magnification). (**a**) Pathologist 2 was unable to make an accurate diagnosis without the assistance of CS-Bacter due to the low bacterial load in the image. (**b**) The biotechnologist was unable to detect the presence of *H. pylori*, even with support from the algorithm, as sample processing artifacts in the image mimicked bacterial structures and caused confusion.

The algorithm further contributed to the standardization of the diagnostic process, as discrepancies between unaided and algorithm-assisted digital diagnosis only arose in one case (1.67%) for pathologist #1 and pathologist #2 (Figure 7a), three cases (5%) for the pathology resident and the pathology technician, and two cases (3.33%) for the biotechnologist (Figure 7b) (Table 4). Most of these ten discrepant cases involved borderline bacterial loads or suboptimal staining intensity, which made unaided diagnosis of *H. pylori* challenging.

## 4. Discussion

Infection with *H. pylori,* a class 1 carcinogen according to the World Health Organization classification with an approximately 50% worldwide prevalence, is a crucial factor in the development of duodenal or gastric ulcers (up to 10% infected individuals), gastric cancer (up to 3%), and gastric mucosa-associated lymphoid-tissue lymphoma (less than 0.01%) [33]. This implies that approximately 6.2% of all cancer cases diagnosed yearly are related to infection with *H. pylori* [34]. As there is evidence that eradication of the pathogen can reduce the risk of suffering from gastric cancer in healthy infected individuals [35], early and accurate detection of *H. pylori* has become essential for patient management. Although there are no specific *H. pylori* stains available at present, there are several options that allow its detection in gastric biopsy samples. The H&E and Giemsa stains have the advantage of being simple and routinely used in pathology laboratories; however, *H. pylori* identification might be notably influenced by interobserver variability and reduced bacterial load when these stains are employed. The WS silver stain offers higher specificity (90–100%) than the H&E and Giemsa stains (87–90%) [15] and is more cost-effective than IHC methods, making it an excellent option for accurate *H. pylori* diagnosis despite its labor-intensive nature.

The use of AI-based tools is expected to have a remarkable impact in hospital and clinical settings. A recent review on the impact of AI in the interpretation of radiology, endoscopy, and histology images of the GI tract predicts the central role that AI will have in the near future on the management of GI patients, helping to standardize and increase the accuracy of diagnosis (potentially at the cost of the deskilling of specialists) and to deliver more personalized medical care [23]. Histopathological diagnosis of *H. pylori* is a simple procedure in cases presenting a high bacterial load; however, it is also a demanding process that requires an exhaustive and painstaking examination of the whole WSI. Furthermore, in cases with reduced numbers or atypical locations of the bacteria, it can become an even more time-consuming process, highly dependable on the skill of the pathologist. The utilization of our algorithm helped to standardize and remarkably reduce interobserver variability by automatically and efficiently detecting *H. pylori* hot-spot areas in the digitalized gastric samples, thus increasing the accuracy and certainty of the diagnosis. This was further demonstrated by the fact that, out of the 60 samples each participant analyzed, the discrepancy between the unaided and the AI-assisted diagnoses occurred only in 1 case for each of the two pathologists, in 3 cases for each the pathology resident and technician, and in 2 cases for the biotechnologist.

Klein et al. previously reported the development of a decision support algorithm for the detection of *H. pylori* in Giemsa-stained gastric samples [36]. Aided by their algorithm, they analyzed 87 WSIs obtained from gastric biopsy samples stained with the modified Giemsa technique and confirmed to be *H. pylori*-positive by PCR and IHC testing. Analysis of the performance of the algorithm showed that it offered 100% sensitivity compared to 68.4% obtained in microscopic diagnosis, supporting its potential as an automatic screening tool for the detection of *H. pylori*. However, it only achieved a specificity of 66.2%, compared to 92.6% obtained in microscopic diagnosis, thus making necessary the employment of a second detection method (PCR, IHC, etc.) to confirm and validate its results. The study does not analyze the potential impact of the application of the algorithm on diagnostic turnaround times. Zhou et al. also reported the development of a DL-based algorithm for the detection of *H. pylori* in H&E gastric samples [37]. In this study, three experienced GI pathologists independently reviewed a set of 65 *H. pylori*-positive and 65 *H. pylori*-negative WSIs with and without the help of their algorithm. Interestingly, they observed a strong performance of their tool in terms of both accuracy and diagnostic time in the case of *H. pylori*-positive samples. Still, diagnostic uncertainty considerably increased in the case of *H. pylori*-negative samples, resulting in a decrease in the accuracy (81 uncertain diagnoses were made when assisted by the algorithm, compared to 60 in the unassisted process). This led the authors to propose the use of the algorithm as a screening tool rather than a primary diagnostic tool. More recently, Zhong et al. evaluated the performance of two different AI models, Faster R-CNN and YOLO v5, on the identification of *H. pylori* from immunochemically stained samples. Initial tests showed that the YOLO v5 model offered better precision and recall than the Faster R-CNN architecture (0.700 and 0.638 vs. 0.536 and 0.582, respectively). Furthermore, an optimized version of the YOLO v5 algorithm exhibited a 16.7% performance improvement [38]. Liscia et al. also developed a DL-based classifier for the identification of *H. pylori* in WS-stained gastric samples. Their model achieved a precision of 0.772 and a recall of 0.897, correctly diagnosing only 80% of the cases. This somewhat disappointing result might be due to the classifier evaluating the samples independently from their gastric inflammatory status, as explained by the authors [39]. In the efficiency study presented here, two pathologists, a pathology resident, a pathology technician, and a biotechnologist each analyzed 60 digitalized WS-stained gastric samples (including *H. pylori*-positive and -negative samples) with and without the aid of our algorithm. The CS-Bacter tool offered an average precision of 0.923 and an average recall of 0.982 (IoU threshold = 0.5), making it an efficient and accurate tool for the screening of *H. pylori* hot-spot areas. Furthermore, a comparison of unaided digital diagnosis (expert review) with diagnosis assisted by the algorithm showed a drastic reduction in diagnostic turnaround time (between 88.13% and 91.76%). Conversely, Zhou et al. only reported a moderate improvement in diagnostic turnaround time in the *H. pylori*-positive images and a negligible change in the case of *H. pylori*-negative images. However, this result is not comparable to the one observed with our algorithm, as the model developed by Zhou et al. utilizes a patch classification method, thus forcing the pathologist to manually verify the patches, while CS-Bacter identifies the exact location of the bacteria, therefore allowing much more efficient detection of *H. pylori.*

The worldwide shortage of expert pathologists is a bottleneck that increases diagnostic turnaround times. A recent Delphi study on the role of AI in pathology in the next decade [40] has predicted the progressive automation of the pathology laboratories, with some tasks (e.g., identification of hot-spot areas, quantification of IHC) routinely performed with the aid of specific AI tools. This study, which included an international panel of experts with first-hand experience in the development and evaluation of AI pathology tools, also predicted a drastic change in the daily work of pathology technicians due to AI adoption, with them routinely performing tasks integrated into digital or computational workflows (e.g., scanner operation, device calibration). However, although most of the experts thought that pathology technicians would be directly involved in making AI-assisted diagnoses, the panel failed to reach a general consensus on this particular point. The efficient automatic detection of *H. pylori* in digitalized gastric biopsy samples by our algorithm and the other models described in this discussion are proof that this predicted automation of the pathology laboratories is already on its way. Furthermore, the efficiency study described here shows that, with some basic training and the aid of our algorithm, the pathology technician and the biotechnologist were able to diagnose the presence of *H. pylori* as efficiently and quickly as the pathology resident (AI-assisted diagnostic turnaround times were 10.53 min for the pathology technician, 10.22 min for the biotechnologist, and 9.83 min for the pathology resident) and almost as quickly as experienced expert pathologists (8.53 min for pathologist #1 and 8.07 min for pathologist #2). In addition, we detected a clear improvement in diagnostic accuracy for the pathology resident (95.0% to 98.3%), pathology technician (93.3% to 98.3%), and biotechnologist (94.2% to 98.3%), presumably because the AI overlay pointed them to specific suspicious structures, thus reducing the search effort and the difficulty of differentiating *H. pylori* from other debris or artifacts.

Nonetheless, this study has some limitations. First, we only included five individuals (two pathologists, a pathology resident, a pathology technician, and a biotechnologist), which restricts the generalizability of the turnaround-time conclusions. Second, we solely focused on WS-stained samples; a more thorough comparison with H&E- or Giemsa-stained samples would be beneficial. Third, despite the large overall sample size for algorithm training, only 300 slides were evaluated in the time efficiency study. Finally, the borderline or suboptimally stained cases represented a challenge even for our algorithm, leading to some diagnostic discrepancies. Future improvements will seek to refine the performance of our algorithm in such challenging situations.

## 5. Conclusions

We have developed a DL-based algorithm, CS-Bacter, that allows the accurate, swift, and efficient detection of *H. pylori* hot spots in digitalized WS-stained gastric samples. Utilization of this algorithm helped to drastically reduce diagnostic turnaround times (between 88.13% and 91.76%, *p* < 0.001). In addition, it improved the accuracy of *H. pylori* detection, particularly for less experienced personnel, by automatically highlighting suspicious areas and reducing the subjectivity of manual searches (use of CS-Bacter increased overall diagnostic accuracy from 95.0% to 98.3% for the pathology resident, from 93.3% to 98.3% for the pathology technician, and from 94.2% to 98.3% for the biotechnologist). Thus, our study demonstrates that, with some basic prior training and the aid of the algorithm, health professionals other than expert pathologists can accurately diagnose *H. pylori* infection, therefore opening the possibility of new roles for the technical healthcare staff. This was evidenced by our observation that, with the assistance of our algorithm, a pathology technician with no prior diagnostic experience was able to diagnose *H. pylori* infection with almost the same accuracy and swiftness as an expert pathologist. We believe that implementation of our algorithm in the pathology workflows will not only considerably reduce diagnostic turnaround times but also offer invaluable support for the diagnosis of uncertain cases.

## Figures and Tables

**Table 1:** Key hyperparameters for the CS-Bacter algorithm.

Parameter	Value
Patch size	1024 × 1024 px
Batch size	32
Optimizer	Adam
Learning rate	0.0001
Training epochs	1500
Loss function	Binary Cross-Entropy

**Table 2:** Description of the different datasets employed in this research.

Purpose	Samples	Description
Training	2640	Manually annotated by expert pathologists. Collected at the HT Medica AP centers.
Calibration (general)	528	To evaluate general performance of the algorithm. Collected at the HT Medica AP centers.
Validation	132	For model threshold adjustment. Collected at the HT Medica AP centers.
Calibration (efficiency study)	100	20 images employed for iterative parameter adjustment and 80 images employed for testing. Collected at the UPIGAP.
Personnel training	200	Training of the pathology technician and the biotechnologist in the identification of *H. pylori* in gastric samples. Extracted from the algorithm training dataset.Collected at the HT Medica AP centers.
Efficiency study	300	150 *H. pylori*-positive and 150 *H. pylori*-negative images to test the proficiency of the CS-Bacter algorithm. Collected at the UPIGAP.

Abbreviations: AP—anatomical pathology; UPIGAP—Unidad Provincial Intercentros de Anatomía Patológica de la Provincia de Granada (Anatomic Pathology Inter-Center Provincial Unit of Granada).

**Table 3:** Traditional vs. AI-assisted digital pathology diagnosis response times.

	Samples	Time		Diagnostic Discrepancy
		DP	AI-Assisted DP	
Pathologist 1	60	4318 s (71.97 min)	512 s (8.53 min)	1 case
Pathologist 2	60	4077 s (67.95 min)	484 s (8.07 min)	1 case
Pathology Resident	60	5891 s (98.18 min)	590 s (9.83 min)	3 cases
Pathology Technician	60	7103 s (118.38 min)	632 s (10.53 min)	3 cases
Biotechnologist	60	7437 s (123.95 min)	613 s (10.22 min)	2 cases

Abbreviations: DP—digital pathology.

**Table 4:** Diagnostic accuracy of the participants with and without AI assistance.

	Diagnostic Accuracy	
	Without AI assistance	With AI assistance
Pathologist 1	98.3%	100%
Pathologist 2	98.3%	100%
Pathology Resident	95.0%	98.3%
Pathology Technician	93.3%	98.3%
Biotechnologist	94.2%	98.3%

Abbreviations: AI—artificial intelligence.

## Data Availability

The data used in this study are not openly available due to commercial and proprietary constraints. Access to the data may be granted by the corresponding author upon reasonable request and is subject to confidentiality agreements.

## References

[1] Robin Warren J., Marshall B. (1983). Unidentified Curved Bacilli on Gastric Epithelium in Active Chronic Gastritis. Lancet.

[2] Mignon M. (2005). The Nobel Prize in Medicine, 2005. Barry J. Marshall and J. Robin Warren. Helicobacter pylori Honored. Med. Sci..

[3] López-Brea M. (2005). La Infección Por *Helicobacter pylori*: Premio Nobel de Medicina. Rev. Esp. Quimioterapia.

[4] Reshetnyak V.I., Burmistrov A.I., Maev I.V. (2021). *Helicobacter pylori*: Commensal, Symbiont or Pathogen?. World J. Gastroenterol..

[5] Gisbert J.P. (2000). A Critical Review of the Diagnostic Methods for *Helicobacter pylori* Infection. Gastroenterol. Hepatol..

[6] Gravina A.G., Zagari R.M., De Musis C., Romano L., Loguercio C., Romano M. (2018). *Helicobacter pylori* and Extragastric Diseases: A Review. World J. Gastroenterol..

[7] Crowe S.E. (2019). *Helicobacter pylori* Infection. N. Engl. J. Med..

[8] Salama N.R., Hartung M.L., Müller A. (2013). Life in the Stomach: Persistence Strategies *Helicobacter pylori*. Nat. Rev. Microbiol..

[9] de Brito B.B., da Silva F.A.F., Soares A.S., Pereira V.A., Santos M.L.C., Sampaio M.M., Neves P.H.M., de Melo F.F. (2019). Pathogenesis and Clinical Management of *Helicobacter pylori* Gastric Infection. World J. Gastroenterol..

[10] Kayali S., Manfredi M., Gaiani F., Bianchi L., Bizzarri B., Leandro G., Di Mario F., De’angelis G.L. (2018). *Helicobacter pylori*, Transmission Routes and Recurrence of Infection: State of the Art. Acta Biomed..

[11] Wang Y.K., Kuo F.C., Liu C.J., Wu M.C., Shih H.Y., Wang S.S.W., Wu J.Y., Kuo C.H., Huang Y.K., Wu D.C. (2015). Diagnosis of *Helicobacter pylori* Infection: Current Options and Developments. World J. Gastroenterol..

[12] Fallone C.A., Chiba N., van Zanten S.V., Fischbach L., Gisbert J.P., Hunt R.H., Jones N.L., Render C., Leontiadis G.I., Moayyedi P. (2016). The Toronto Consensus for the Treatment of *Helicobacter pylori* Infection in Adults. Gastroenterology.

[13] Smith S.M., O’Morain C., McNamara D. (2019). *Helicobacter pylori* Resistance to Current Therapies. Curr. Opin. Gastroenterol..

[14] Cardos A.I., Maghiar A., Zaha D.C., Pop O., Fritea L., Miere F., Cavalu S. (2022). Evolution of Diagnostic Methods for *Helicobacter pylori* Infections: From Traditional Tests to High Technology, Advanced Sensitivity and Discrimination Tools. Diagnostics.

[15] Lee J.Y., Kim N. (2015). Diagnosis of *Helicobacter pylori* by Invasive Test: Histology. Ann. Transl. Med..

[16] Mejía-Parra J.L.J., Guerrero-Espinoza A.E., Flores-Arrascue C.P., Chiclayo-Padilla A.S. (2020). Estandarización de Nuevo Protocolo Inmunohistoquímico Para Identificar *Helicobacter pylori* de Biopsias Gástricas y Valoración Frente a La Tinción Hematoxilina-Eosina. Rev. Cuerpo Médico Hosp. Nac. Almanzor Aguinaga Asenjo.

[17] Ashton-Key M., Diss T.C., Isaacson P.G. (1996). Detection of *Helicobacter pylori* in Gastric Biopsy and Resection Specimens. J. Clin. Pathol..

[18] Akeel M., Elhafey A., Shehata A., Elmakki E., Aboshouk T., Ageely H., Mahfouz M.S. (2021). Efficacy of Immunohistochemical Staining in Detecting *Helicobacter pylori* in Saudi Patients with Minimal and Atypical Infection. Eur. J. Histochem..

[19] Ginestet F., Guibourg B., Doucet L., Théreaux J., Robaszkiewicz M., Marcorelles P., Uguen A. (2017). Upfront Immunohistochemistry Improves Specificity of *Helicobacter pylori* Diagnosis. A French Pathology Laboratory Point of View. Helicobacter.

[20] Benoit A., Hoyeau N., Fléjou J.F. (2018). Diagnosis of *Helicobacter pylori* Infection on Gastric Biopsies: Standard Stain, Special Stain or Immunohistochemistry?. Ann. Pathol..

[21] Jonkers D., Stobberingh E., De Bruine A., Arends J.W., Stockbrügger R. (1997). Evaluation of Immunohistochemistry for the Detection of *Helicobacter pylori* in Gastric Mucosal Biopsies. J. Infect..

[22] Skrebinska S., Megraud F., Daugule I., Santare D., Isajevs S., Liepniece-Karele I., Bogdanova I., Rudzite D., Vangravs R., Kikuste I. (2022). Who Could Be Blamed in the Case of Discrepant Histology and Serology Results for *Helicobacter pylori* Detection?. Diagnostics.

[23] Berbís M.A., Aneiros-Fernández J., Olivares F.J.M., Nava E., Luna A. (2021). Role of Artificial Intelligence in Multidisciplinary Imaging Diagnosis of Gastrointestinal Diseases. World J. Gastroenterol..

[24] Esteva A., Robicquet A., Ramsundar B., Kuleshov V., DePristo M., Chou K., Cui C., Corrado G., Thrun S., Dean J. (2019). A Guide to Deep Learning in Healthcare. Nat. Med..

[25] Topol E.J. (2019). High-Performance Medicine: The Convergence of Human and Artificial Intelligence. Nat. Med..

[26] Choi R.Y., Coyner A.S., Kalpathy-Cramer J., Chiang M.F., Peter Campbell J. (2020). Introduction to Machine Learning, Neural Networks, and Deep Learning. Transl. Vis. Sci. Technol..

[27] Serajian M., Testagrose C., Prosperi M., Boucher C. (2025). A Comparative Study of Antibiotic Resistance Patterns in *Mycobacterium tuberculosis*. Sci. Rep..

[28] Irani H., Metsis V. (2024). Enhancing Time-Series Prediction with Temporal Context Modeling: A Bayesian and Deep Learning Synergy. Int. Flairs Conf. Proc..

[29] Tan M., Le Q.V. EfficientNet: Rethinking Model Scaling for Convolutional Neural Networks. Proceedings of the 36th International Conference on Machine Learning.

[30] Wang J., Zhang X., Lv P., Zhou L., Wang H. (2021). EAR-U-Net: EfficientNet and Attention-Based Residual U-Net for Automatic Liver Segmentation in CT. arXiv.

[31] He K., Zhang X., Ren S., Sun J. (2016). Deep Residual Learning for Image Recognition. Proceedings of the 2016 IEEE Conference on Computer Vision and Pattern Recognition (CVPR).

[32] Lin T.-Y., Dollár P., Girshick R., He K., Hariharan B., Belongie S. Feature Pyramid Networks for Object Detection. Proceedings of the IEEE Conference on Computer Vision and Pattern Recognition.

[33] McColl K.E.L. (2010). *Helicobacter pylori* Infection. N. Engl. J. Med..

[34] Plummer M., Franceschi S., Vignat J., Forman D., De Martel C. (2015). Global Burden of Gastric Cancer Attributable to *Helicobacter pylori*. Int. J. Cancer.

[35] Ford A.C., Yuan Y., Forman D., Hunt R., Moayyedi P. (2020). *Helicobacter pylori* Eradication for the Prevention of Gastric Neoplasia. Cochrane Database Syst. Rev..

[36] Klein S., Gildenblat J., Ihle M.A., Merkelbach-Bruse S., Noh K.W., Peifer M., Quaas A., Büttner R. (2020). Deep Learning for Sensitive Detection of *Helicobacter pylori* in Gastric Biopsies. BMC Gastroenterol..

[37] Zhou S., Marklund H., Blaha O., Desai M., Martin B., Bingham D., Berry G.J., Gomulia E., Ng A.Y., Shen J. (2020). Deep Learning Assistance for the Histopathologic Diagnosis of *Helicobacter pylori*. Intell. Based Med..

[38] Zhong Z., Wang X., Li J., Zhang B., Yan L., Xu S., Chen G., Gao H. (2022). A Study on the Diagnosis of the *Helicobacter pylori* Coccoid Form with Artificial Intelligence Technology. Front. Microbiol..

[39] Liscia D.S., D’Andrea M., Biletta E., Bellis D., Demo K., Ferrero F., Petti A., Butinar R., D’Andrea E., Davini G. (2022). Use of Digital Pathology and Artificial Intelligence for the Diagnosis of *Helicobacter pylori* in Gastric Biopsies. Pathologica.

[40] Berbís M.A., McClintock D.S., Bychkov A., Van der Laak J., Pantanowitz L., Lennerz J.K., Cheng J.Y., Delahunt B., Egevad L., Eloy C. (2023). Computational Pathology in 2030: A Delphi Study Forecasting the Role of AI in Pathology Within the next Decade. eBioMedicine.

